# The involvement of soluble epoxide hydrolase in the development of cardiovascular diseases through epoxyeicosatrienoic acids

**DOI:** 10.3389/fphar.2024.1358256

**Published:** 2024-04-02

**Authors:** Shan Jiang, Siyi Han, Dao Wen Wang

**Affiliations:** ^1^ Division of Cardiology, Department of Internal Medicine, Tongji Hospital, Tongji Medical College, Huazhong University of Science and Technology, Wuhan, China; ^2^ Hubei Key Laboratory of Genetics and Molecular Mechanisms of Cardiological Disorders, Wuhan, China; ^3^ Department of Anesthesiology and Pain Medicine, Hubei Key Laboratory of Geriatric Anesthesia and Perioperative Brain Health, Wuhan Clinical Research Center for Geriatric Anesthesia, Tongji Hospital, Tongji Medical College, Huazhong University of Science and Technology, Wuhan, China

**Keywords:** epoxyeicosatrienoic acids, soluble epoxide hydrolase, myocardial infarction, coronary heart disease, hypertension, heart failure

## Abstract

Arachidonic acid (AA) has three main metabolic pathways: the cycloxygenases (COXs) pathway, the lipoxygenases (LOXs) pathway, and the cytochrome P450s (CYPs) pathway. AA produces epoxyeicosatrienoic acids (EETs) through the CYPs pathway. EETs are very unstable *in vivo* and can be degraded in seconds to minutes. EETs have multiple degradation pathways, but are mainly degraded in the presence of soluble epoxide hydrolase (sEH). sEH is an enzyme of bifunctional nature, and current research focuses on the activity of its C-terminal epoxide hydrolase (sEH-H), which hydrolyzes the EETs to the corresponding inactive or low activity diol. Previous studies have reported that EETs have cardiovascular protective effects, and the activity of sEH-H plays a role by degrading EETs and inhibiting their protective effects. The activity of sEH-H plays a different role in different cells, such as inhibiting endothelial cell proliferation and migration, but promoting vascular smooth muscle cell proliferation and migration. Therefore, it is of interest whether the activity of sEH-H is involved in the initiation and progression of cardiovascular diseases by affecting the function of different cells through EETs.

## 1 Introduction

**FIGURE 1:**
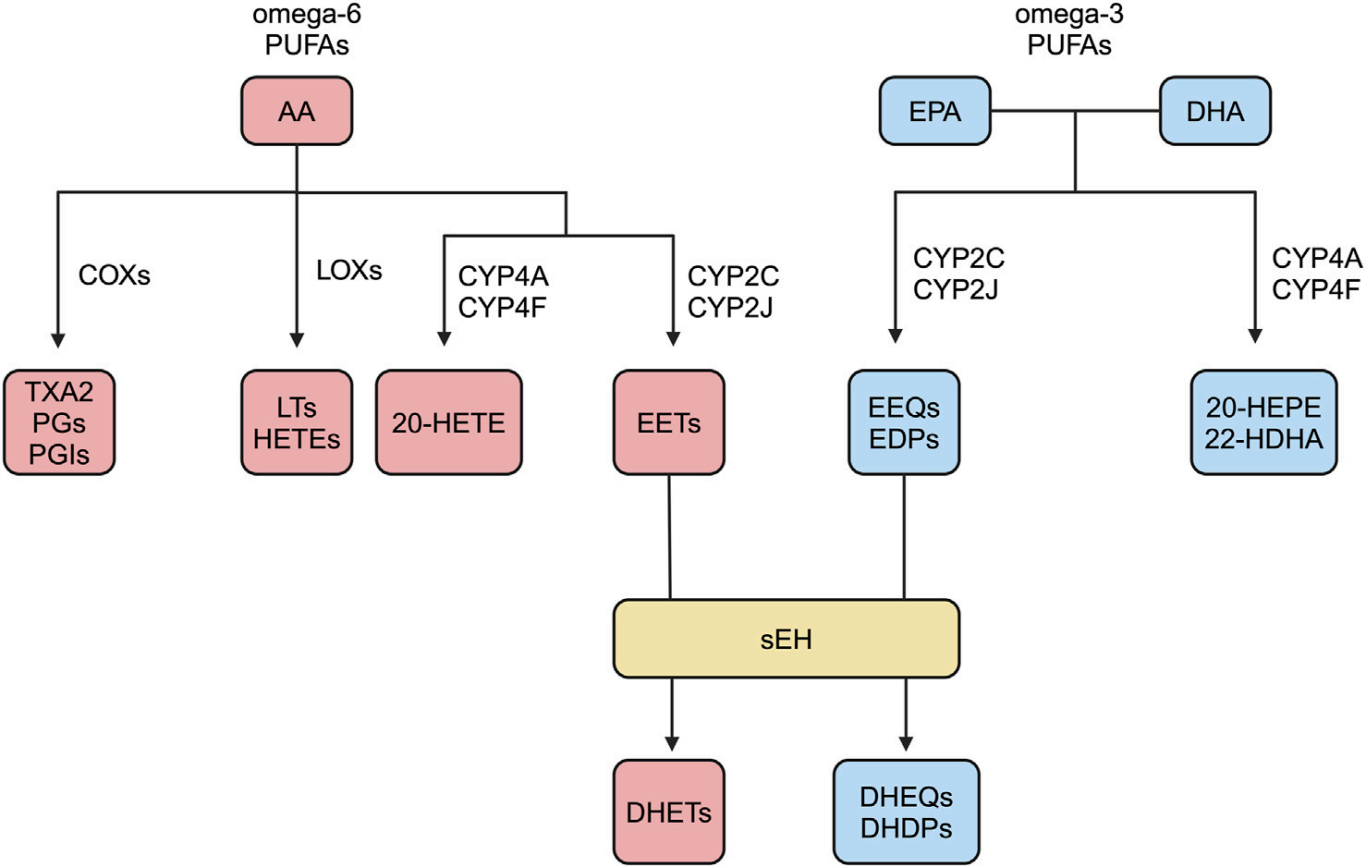
Overview of omega-3 and omega-6 PUFAs metabolic pathways.

In response to stimuli such as inflammation and injury, cell membrane phospholipids release free AA in the presence of activated phospholipase A2 (Wang et al., 2020; Shi et al., 2022). Polyunsaturated fatty acids (PUFAs) are mainly categorized as omega-3 and omega-6, as well as the less common omega-4 and omega-7 (Atone et al., 2020; Pallas et al., 2020; Saito, 2008). AA has many metabolic pathways. AA undergo COXs pathway, by giving rise to TXA2, PGs, and PGIs; LOXs pathway, by giving rise to LTs and HETEs; CYPs pathway, by giving rise to EETs and HETEs (Panigrahy et al., 2010; Wang et al., 2018; Imig, 2019; Ren, 2019; Hoxha et al., 2020; Shan and Hashimoto, 2022; Zhang et al., 2022). EETs are mainly produced by endothelial cells and cardiomyocytes, and they have a short half-life and are rapidly degraded *in vivo* (Wang and Dubois, 2012). Membrane receptors of EETs include prostaglandin E receptor2 (EP2), thromboxane receptor (TP), etc., and EP2 and TP belong to G-protein coupled receptors (GPCR) (Behm et al., 2009; Yang et al., 2010). It has been reported in the literature that EETs require G protein signaling components for vasodilatory effects (Li and Campbell, 1997; Fukao et al., 2001). However, the putative high-affinity G-protein coupled EET receptor has not been identified. EETs have multiple degradation pathways, such as β-oxidation to form smaller reactive epoxides, binding to fatty acid binding protein, and binding to cell membrane phospholipids, but mainly catalyzed by the activity of sEH-H to generate corresponding diols with no or low biological activity, namely, dihydroxyeicosatrienoic acids (DHETs) (Figure 1) (Zhao et al., 2012; Askari et al., 2013; Davis et al., 2017; Swardfager et al., 2018).

Mammalian EHs can be categorized as sEH, microsomal epoxide hydrolase (mEH), cholesterol EH (ChEH), leukotriene A4 hydrolase (LTA4H), and hepoxilin EH (Morisseau and Hammock, 2013; Edin and Zeldin, 2021). sEH and mEH are two of the most studied EHs. Different EHs catalyze different substrates. sEH mainly catalyzes endogenous EpFAs, mEH mainly catalyzes toxic xenobiotic derived epoxides, LTA4H mainly catalyzes leukotriene A4, ChEH mainly catalyzes cholesterol-5,6-epoxide, hepoxilin EH mainly catalyzes hepoxilin (Newman et al., 2005; Decker et al., 2009). Thus *in vivo* EETs are mainly hydrolyzed catalytically by sEH. Human mEH is encoded by the EPHX1 gene; human sEH is encoded by the EPHX2 gene, located on the short arm of chromosome 8 (8p21-p12) (Pillarisetti and Khanna, 2015; Liu, 2018; Sun et al., 2021). sEH is widely distributed in the body, such as liver, kidney, lungs, brain, heart, spleen, intestines, etc., but is mainly located in the liver and kidney (Wang Y. X. et al., 2010; Hashimoto, 2019). sEH is mainly located in the cytoplasm of cells and peroxisomes, and is a homodimer composed of two monomers with a molecular weight of 60 kDa, each with two functionally distinct structural domains (Harris and Hammock, 2013; He et al., 2016; Hu et al., 2018; Jones et al., 2019; Zarriello et al., 2019). The N-terminal structural domain has lipid phosphatase (sEH-P) activity, which catalyzes the hydrolysis of lysophosphatidic acids, and the C-terminal structural domain has sEH-H activity, which catalyzes the hydrolysis of EETs to DHETs, thus sEH is a bifunctional enzyme (Morisseau et al., 2012; Huang et al., 2016; Kramer and Proschak, 2017; Domingues et al., 2019). Peroxisome proliferator-activated receptor gamma (PPARγ) agonists such as troglitazone and rosiglitazone exert opposing effects on the regulation of sEH expression in different tissues. In adipose tissue, sEH expression is upregulated, but in cardiomyocytes, sEH expression is downregulated (Pang et al., 2011). AngII can also promote sEH expression. In vascular endothelial cells, AngII promotes the binding of the transcription factor c-Jun binding to the promoter region of the sEH gene, thereby upregulating sEH expression (Ai et al., 2007).

Cardiovascular diseases stand as the foremost cause of global mortality, mainly including myocardial infarction, coronary heart disease, hypertension, cardiac hypertrophy, and heart failure (Evangelista et al., 2018). Inhibiting sEH activity or gene deletion encoding sEH can reduce the degradation of EETs, prolong their half-life, increase endogenous EET levels, and enhance their cardiovascular protective effects, including vasodilation, anti-inflammatory, lipid-lowering, and anti-apoptosis effects (Qiu et al., 2011; Romashko et al., 2016; Wang et al., 2021; Shi et al., 2022). Consequently, sEH emerges as a prospective therapeutic target for cardiovascular diseases. Current research on sEH has mainly focused on its sEH-H activity. As mentioned earlier, the activity of sEH-H assumes a crucial role by degrading EETs and inhibiting their effects. Therefore, we would like to understand the role of EETs in different cells and how they are involved in influencing the development of cardiovascular diseases.

### 1.1 Myocardial infarction

Clinical studies have shown that plasma levels of EETs and DHETs correlate with infarction rates, with elevated plasma EETs associated with low infarction rates, but elevated plasma DHETs associated with high infarction rates (Lemaitre et al., 2022). In a rat model of myocardial infarction, oral ACE inhibitors reduce the production of AngII and exert cardioprotective effects (Tanonaka et al., 1996). AngI generates AngII under the catalysis of ACE and chymase (mainly derived from mast cells) (Froogh et al., 2020). AngII receptors mainly include ANG II subtype 1 (AT1) and ANG II subtype 2 (AT2) (Tsuchiya et al., 1997). Previous studies have shown that activation of ATP-sensitive K+ channel (KATP) can reduce the area of myocardial infarction in animals and exert cardioprotective effects (Gross and Auchampach, 1992). KATP is expressed in many tissues, such as cardiomyocytes, vascular smooth muscle cells, and pancreatic beta cells (Yokoshiki et al., 1998). There are two types of KATP in cardiomyocytes: sarcolemmal KATP (sarcKATP) channels and mitochondrial KATP (mitoKATP) channels (Hu et al., 1999). Elevated intracellular ATP inhibits KATP channels (Lu et al., 2006). In guinea-pig venturar myocyte, AngII inhibited KATP activity by binding to the AT1 receptor and elevating ATP levels in the subsarcolemmal (Tsuchiya et al., 1997). It suggests that increased AngII is detrimental to the recovery of cardiac function after myocardial infarction. In myocardial infarction, the cardioprotective effect of KATP is mainly derived from the activation of mitoKATP rather than sarcKATP (Liu et al., 1998). Application of sEH null mice and sEH inhibitor tAUCB showed that inhibition of sEH improved mitochondrial function in the infarcted area of the mouse heart and exerted cardioprotective effect (Akhnokh et al., 2016; Jamieson et al., 2017). This protective effect stems mainly from the fact that both knockdowns of sEH and inhibition of sEH activity increase the levels of EETs in mouse plasma, leading to the activation of mitoKATP, which is abrogated by application of the mitoKATP channel inhibitor 5-HD (Seubert et al., 2006). MitoKATP activation can promote mitochondrial K+ influx, inhibit calcium ion entry into mitochondria, and alleviate mitochondrial calcium overload (Murata et al., 2001). Mitochondrial calcium overload will stimulate the opening of mitochondrial permeability transition pore (MPTP) and leak cytochrome c into the cytoplasm, resulting in mitochondrial swelling, reduced ATP production, and mitochondrial dysfunction, eventually leading to apoptosis (Brookes et al., 2004; Gross et al., 2013). EETs activate the cardiomyocyte mitoKATP by activating the mitogen-activated protein kinase (MAPK) signaling pathway and protect cardiomyocytes (Seubert et al., 2004). EETs also reduce the sensitivity of mitoKATP to intracellular ATP in cardiomyocytes, allowing mitoKATP to open at normal ATP concentrations (Lu et al., 2001). Taken together, this suggests that EETs maintain mitochondrial function and inhibit cardiomyocyte apoptosis through activation of mitoKATP (Bodiga et al., 2009; Katragadda et al., 2009). Cardiomyocyte apoptosis plays an important role in the onset and progression of myocardial infarction (Marinovic et al., 2008). Exogenous supplementation of EET reduces cardiomyocyte apoptosis and decreases infarct size in mouse heart tissue (Bodiga et al., 2009). In HL1 and primary neuronal cardiomyocytes, EETs increase the intracellular levels of anti-apoptotic protein X-linked inhibitor of apoptosis (XIAP) and decrease the activity of pro-apoptotic proteases caspase-3 and caspase-9 by activating the phosphatidylinositol 3 (PI3)-kinase/Akt pathways, inhibiting cardiomyocyte apoptosis (Dhanasekaran et al., 2008).

Angiogenesis restores blood supply to the infarcted area of the heart, protects cardiac function, and is an important treatment for myocardial infarction (Cochain et al., 2013). Endothelial progenitor cells, which originate from bone marrow and non-bone marrow organs such as the spleen, can proliferate and differentiate into mature vascular endothelial cells (Hristov and Weber, 2008; Zampetaki et al., 2008). The proliferation and migration of endothelial cells lead to nascent capillary structure formation, promoting angiogenesis (Pozzi et al., 2005).

EETs can exert pro-angiogenic effects by promoting the function of endothelial progenitor cells (EPCs): previous literature has reported that miR-126 can promote the recruitment of bone marrow-derived EPCs to the infarcted area of the heart by inducing the release of chemokine CXCL12 or stromal cell-derived factor-1 (SDF-1) from endothelial cells in the infarcted area (van Solingen et al., 2011). sEH inhibitor TPPU activates the AKT/GSK3βpathway or ERK/p38 MAPK signaling pathway by elevating the level of EETs, promoting miR-126 expression, enhancing the recruitment of bone marrow-derived EPCs to the infarcted area, which in turn promotes angiogenesis and post-MI cardiac repair (Guo et al., 2018; Gui et al., 2020). Key pro-angiogenic growth factors, such as vascular endothelial growth factor (VEGF) and basic fibroblast growth factor (bFGF), play vital roles (Virag et al., 2007; Oguro et al., 2009). EETs exert a pro-angiogenic effect by promoting the expression of VEGF and bFGF (Zhao et al., 2018). In human dermal microvascular endothelial cells (HDMVEC), 14,15-EET promotes the expression and release of bFGF, and the application of bFGF neutralizing antibodies can block the promoting effect of 14,15-EET on angiogenesis (Zhang et al., 2006). sEH inhibitor AUDA can promote the proliferation of human coronary arterial endothelial cells (HCAECs) by upregulating PPARγ (Dai et al., 2020). sEH inhibitor tAUCB activates PPARγ by elevating the levels of EETs, which promotes endothelial cell migration and angiogenesis by promoting the expression of the VEGF and hypoxia-inducible factor-1alpha (HIF-1a) (Xu et al., 2013). HIF-1a is the main upstream molecule that promotes VEGF expression (Tsuzuki et al., 2000). In HCAECs, EETs play a role in promoting angiogenesis by elevating VEGF expression through promoting HIF-1a expression (Gui et al., 2022). In Bovine aortic endothelial cells (BAECs), EETs promote endothelial cell proliferation, migration, and angiogenesis through MAPK and PI3K/Akt pathways, reducing infarct size (Wang et al., 2005). Thus, VEGF binds to receptors on endothelial cells and activates PI3K/Akt and MAPK signaling pathways in endothelial cells to exert pro-angiogenic effects (Yang et al., 2009).

sEH-P can also be involved in the occurrence and development of myocardial infarction. The sEH-P knock-in animal experiments showed that sEH-P could reduce the area of myocardial infarction, and promote the recovery of myocardial contractile function, which was beneficial to prognosis (Leuillier et al., 2023). The mechanism is not yet clear.

### 1.2 Atherosclerosis

Atherosclerosis, a chronic inflammatory condition, manifests as the formation of lipid plaques within the arterial wall (Dunn et al., 2008). Atherosclerosis occurring in the coronary arteries can cause coronary heart disease. Previous studies have shown that oral inhibitors of sEH can reduce the size of atherosclerotic plaques and slow the progression of atherosclerosis (Zhang et al., 2009). Clinical studies have shown that the CYP metabolic pathway of AA is disturbed in patients with coronary heart disease, and their plasma 14,15-DHET levels are increased (Theken et al., 2012; Yang et al., 2013).

Elevated plasma oxidatively modified low density lipoprotein-cholesterol (Ox-LDL) levels and endothelial cell injury are independent risk factors for atherosclerosis (Vink et al., 2000; Geng et al., 2010). Ox-LDL binds to the endothelium-expressed scavenger receptor lectin-like oxidized low-density lipoprotein receptor-1 (LOX-1) leads to endothelial cell injury, including endothelial cell dysfunction and increased apoptosis (Zhang et al., 2012). Endothelial cell dysfunction activates NF-κB, leading to increased expression of endothelial cell surface adhesion molecules such as P-selectin, E-selectin, vascular cell adhesion molecule (VCAM-1), intercellular adhesion molecule (ICAM-1), and monocyte chemotactic protein (MCP-1), monocytes in the circulating blood flow adhere to the surface of endothelial cells and migrate to the subendothelium (Mitra et al., 2011). The increased apoptosis of endothelial cells was due to increased Bax expression, decreased Bcl expression and increased Bax/Bcl ratio (Li and Mehta, 2000). Ox-LDL can upregulate the expression of LOX-1 in endothelial cells (Jiang et al., 2014). In rat pulmonary artery endothelial cells (RPAECs), EETs reduce the upregulation of ICAM-1, E-selectin, and MCP-1 expression caused by Ox-LDL by inhibiting the upregulation of LOX-1 expression and NF-kB activation (Jiang et al., 2015). In BAECs, 11,12-EET inhibits the expression of endothelial cell adhesion molecules VCAM-1, E-selectin, and ICAM-1 by suppressing NF-kB activation, which in turn reduces monocyte adhesion (Node et al., 1999; Sun et al., 2016). P-selectin glycoprotein ligand 1 (PSGL-1) can be expressed on blood monocytes and is a ligand for P-, E−, and L-selectins (Huo and Xia, 2009). sEH inhibitor t-TUCB and 14,15-EET inhibit monocyte adhesion to endothelial cells by decreasing blood peripheral blood mononuclear cells (PBMC) PSGL-1 expression and monocyte PSGL-1 binding to endothelial cell P and E-selectin (Li D. et al., 2016). 11,12-EET exerts anti-endothelial cell apoptosis by promoting AKT1 activation, which inhibits the expression of the pro-apoptotic protein BIM by repressing the transcription factor FOXO1 (Liu et al., 2016). 14,15-EET promotes the expression of the anti-apoptotic proteins Bcl-2 and Bcl-xL, while inhibiting the expression of the pro-apoptotic protein Bax expression thereby inhibiting endothelial cell apoptosis (Feng et al., 2013). In brain microvascular endothelial cells, 14,15-EET inhibited endothelial cell apoptosis by inhibiting Rho-kinase (ROCK) activation (Gupta et al., 2012). Previous studies have shown that ROCK induces endothelial cell apoptosis by inhibiting PI3K/Akt activity (van der Heijden et al., 2008). It suggests that EETs inhibit endothelial cell apoptosis through the activation of the PI3K/Akt signaling pathway.

Circulating monocytes that migrate to the subendothelium can differentiate into macrophages (Ross, 1999). Foam cells, pivotal in atherosclerosis development, play a crucial role, and inhibition of foam cell formation attenuates atherosclerosis in experimental animal models (Tsai et al., 2010). The formation of foam cells includes increased cholesterol uptake and impaired cholesterol efflux. Macrophages recognize and uptake Ox-LDL through scavenger receptors (SRs) such as SR-A class, CD36, and LOX-1 (Rahaman et al., 2006). Macrophage reverse cholesterol transports (RCTs) such as SR-BI, ATP-binding cassette transporter 1 (ABCA1), and ATP-binding cassette sub-family G member-1 (ABCG1) mediate cholesterol efflux injury (Maguire et al., 2019). The combined effect leads to the accumulation of large amounts of lipoprotein-derived cholesterol in macrophages, forming foam cells. ABCA1 is degraded in the presence of calpain protease (Martinez et al., 2003). Previous literature reported that in THP-1 haem oxygenase-1 (HO-1) inhibits transcription factor AP-1-mediated SR-A expression and decreases calpain protease activity to increase ABCA1 stability (Tsai et al., 2010). TPPU inhibits the EH activity of sEH, stabilizes EETs, enhances the role of EETs in promoting HO-1 expression, and finally inhibits the formation of foam cells through HO-1 mediated cholesterol regulation (Peng et al., 2019). Vascular smooth muscle cells can also form foam cells by uptake of cholesterol (Allahverdian et al., 2014). Therefore, the migration and proliferation of vascular medial smooth muscle cells to the subendothelium assume a crucial role in atherosclerotic plaque formation (Raines and Ross, 1993). Cyclin D1 is a downstream molecule of the MAPK signaling pathway (Lavoie et al., 1996). In human aortic smooth muscle cells, the sEH inhibitor CDU did not affect the activation of the MAPK signaling pathway but instead directly decreased the level of cyclin D1 and inhibited the proliferation of vascular smooth muscle cells (Feng et al., 2013). In rat aortic smooth muscle cells, 5,6-, 11,12-, and 14,15 -EETs increase intracellular cAMP levels and protein kinase A (PKA) activity downstream of cAMP and the application of cAMP and PKA inhibitors reversed the inhibitory effects of EETs on smooth muscle cell migration, suggesting that EETs inhibit smooth muscle cell migration through activation of the cAMP/PKA pathway (Sun et al., 2002). Previous studies have shown that the cAMP/PKA pathway regulates smooth muscle cell migration in rat aortic smooth muscle cell and human venous smooth muscle cell, and the application of the cAMP analogue 8-bromo cAMP and the cAMP agonist forskolin exerted inhibitory effects on smooth muscle cell migration (Itoh et al., 2001; Sun et al., 2002).

sEH-P can also be involved in the occurrence and development of atherosclerosis. Reduced sEH-P activity of the Glu287Arg variant in sEH, which is a risk factor for atherosclerosis (Fornage et al., 2004; Purba et al., 2015). Phosphorylated ABCA1 is less stable and hydrolyzed by calpain protease (Martinez et al., 2003). In the mouse model of atherosclerosis, the expression of sEH is elevated in foam cells, and the elevated activity of sEH-P dephosphorylates ABCA1, increases the stability of ABCA1, and enhances cholesterol efflux, thereby attenuating cholesterol accumulation in the macrophage, decreasing foam cell formation, and slowing down the progression of atherosclerosis, at which time the activity of sEH-P plays a protective role in atherosclerosis (Lien et al., 2019).

### 1.3 Hypertension

In the spontaneously hypertensive rat (SHR) model, sEH activity was increased and plasma EETs levels decreased; plasma EETs increased and blood pressure decreased with the use of the sEH inhibitor AUCB (Jiang et al., 2011). In the angiotensin II-induced hypertension model, the expression of sEH in the kidneys of rats increases, and application of the sEH inhibitor NCND decreased blood pressure (Imig et al., 2002).

AngII plays an important role in the development of hypertension. Ang II activates the AT1 receptor, which promotes vasoconstriction and elevates blood pressure (Carroll et al., 2014). In SHR rats and AngII-infused Wistar rats models, Ang II promotes sEH transcription and increases sEH expression in rat heart (Ai et al., 2007). *In vitro* experiments have shown that in endothelial cells Ang II promotes sEH expression through the transcription factor c-Jun (Ai et al., 2007).

EETs can lower blood pressure through the following mechanisms.

Fristly, EETs, as endothelium-derived hyperpolarizing factor (EDHF), activate subunit of heterotrimeric G proteins (Gsα) and downstream cAMP/PKA pathway through GPCR of smooth muscle cells, upregulating intracellular calcium ion concentration ([Ca2+]i), activate smooth muscle cell calcium-dependent potassium channels (KCa), causes K+ channel opening, K+ outflow, and hyperpolarize the arterial smooth muscle cells (Fang et al., 1999; Carroll et al., 2006; Alkayed et al., 2022). As a result, voltage-dependent Ca2 channels are closed, and the concentration of calcium ions in smooth muscle cells is reduced, leading to vasodilation (Kopf et al., 2011; Zhu et al., 2022). Application of KCa inhibitors TEA and charybdotoxin inhibits the vasodilatory effects of EETs (Campbell et al., 1996).

Secondly, EETs activate PKA, leading to the opening of KATP in vascular smooth muscle cells, promoting K + efflux and inhibiting calcium ion influx, resulting in hyperpolarization of vascular smooth muscle cells and vasodilation (Ye et al., 2005).

Thirdly, EETs can promote eNOS expression in BAECs through activation of the MAPK/PKC signaling pathway, and eNOS promotes NO production (Wang et al., 2003). The blood pressure is lowered by the vasodilatory effect of NO (Huang et al., 1995). EDHF and NO are the two main endothelial cell-produced active substances that regulate vascular tone (Bauersachs et al., 1997).

Fourthly, EETs can inhibit renal sodium reabsorption. 5,6-EET inhibits proximal tubular epithelial cell Na-K-ATPase activity (Satoh et al., 1993). 14,15-EET inhibits Na-K-2Cl transport activity in renal epithelial cell line (He et al., 2003). 8,9-EET, 11,12-EET and 14,15-EET inhibit renal cortical collecting duct epithelial Na channel (ENaC) activity by phosphorylating ENaC β and γ subunits (Wei et al., 2004; Pavlov et al., 2011; Pidkovka et al., 2013). The combined effect increases renal sodium excretion and urine volume, and reduces plasma sodium levels, resulting in a compensatory decrease in extracellular fluid volume and a decrease in blood pressure (Capdevila et al., 2014).

Fifthly, EETs indirectly reduces blood pressure through the vasodilation and urinary sodium excretion effects of ANP (Anand-Srivastava, 2005; Nishikimi et al., 2006; Xiao et al., 2010).

Hypertension can develop into cardiac hypertrophy, leading to cardiac systolic and diastolic dysfunction (Xiao et al., 2010). In SHR rats, Ang II-infused Wistar rats and isoprenaline-induced cardiac hypertrophy animal models showed increased expression of sEH in hypertrophied cardiac tissue (Zordoky et al., 2008; Ai et al., 2009b). In the isoprenaline-induced cardiac hypertrophy rat cardiomyoblast cell line (H9c2) cell models, the sEH inhibitors TUPS and 11,12-EET both reduced the expression of isoprenaline-mediated hypertrophic markers atrial natriuretic peptide (ANP), brain natriuretic peptide (BNP), and β-myosin heavy chain (β-MHC) (Althurwi et al., 2013). In the isoproterenol-induced cardiac hypertrophy rat model, EETs reduce the expression of ANP and BNP, as well as the heart weight to body weight ratio (Althurwi et al., 2014). An decrease in the expression of ANP, BNP, and β-MHC and in the heart weight to body weight ratio indicates a reduction in cardiac hypertrophy (Aboutabl et al., 2009). The mechanisms by which EETs reduce cardiac hypertrophy may be as follows. EETs can lower blood pressure, as described previously, and inhibition of sEH can increase the level of EETs, which can in turn reduce cardiac hypertrophy. Autophagy plays an important role in the pathological process of cardiac hypertrophy, and excessive autophagy can exacerbate cardiac hypertrophy (Li L. et al., 2016). Previous studies have shown that the mammalian target of the rapamycin (mTOR) signaling pathway inhibits autophagy in cardiomyocytes (Li et al., 2017). In angiotensin II-induced cardiac hypertrophy H9C2 cell models, the sEH inhibitor TUPS inhibits autophagy by activating the mTOR signaling pathway, reducing the expression of cardiac hypertrophy markers ANP and BNP, and alleviating cardiac hypertrophy (Zhang et al., 2019). Previous studies have shown that bFGF promotes cardiac hypertrophy (Schultz et al., 1999). In Ephx2 (−/−) mouse experiments, sEH deletion reduced bFGF expression in cardiac tissues by inhibiting MAPK activation, thereby alleviating mouse cardiac hypertrophy (Zhang et al., 2014).

sEH-P can also be involved in the development of hypertension. sEH-P inhibits endothelial cell eNOS phosphorylation and reduces eNOS activity, leading to a decrease in NO production by endothelial cells (Hou et al., 2012; Hou et al., 2015). sEH-P may regulate hypertension through this mechanism.

### 1.4 Heart failure

Myocardial infarction, coronary heart disease, hypertension, and cardiac hypertrophy can all develop into heart failure, increasing cardiovascular mortality rate (Carreño et al., 2006; Roger, 2013; El-Sherbeni and El-Kadi, 2014; Cervenka et al., 2018). In a rat model of heart failure, the expression and activity of sEH in cardiac tissue increased, leading to a decrease in EETs levels (161).

The pathogenesis of heart failure includes cardiomyocyte apoptosis, oxidative stress and inflammation (Wencker et al., 2003; Tsutsui et al., 2011; Biasucci et al., 2017).

There are two main types of intracellular organelles that mediate apoptosis. The first is the mitochondria-mediated pathway. As mentioned previously EETs can protect mitochondrial function and inhibit cardiomyocyte apoptosis through activation of mitoKATP (Akao et al., 2001). The second is the endoplasmic reticulum-mediated pathway. Various factors such as increased reactive oxygen species (ROS) production and dysregulation of calcium homeostasis can cause endoplasmic reticulum stress and lead to cardiomyocyte apoptosis (von Harsdorf et al., 1999). The function of sarcoplasmic/ER calcium ATPase (SERCA2a) is to transport intracellular Ca (2+) to the sarcoplasmic reticulum lumen, causing cardiomyocytes relaxation, which requires the consumption of ATP (167). The expression of SERCA2a is downregulated in myocardial tissues of heart failure patients and mice (Hasenfuss et al., 1994; Zarain-Herzberg et al., 1996). Application of gene therapy to restore SERCA2a expression can enhance cardiac function and play a therapeutic role in heart failure patients (Jessup et al., 2011). In H9c2 cells, EETs inhibited endoplasmic reticulum stress-induced cardiomyocyte apoptosis and exerted cardioprotective effects by decreasing [Ca2+]i through increasing the expression of SERCA2a and maintaining Ca2+ homeostasis (Wang et al., 2014; You et al., 2016).

Oxidative stress plays an important role in the pathophysiological process of heart failure and is involved in the development of heart failure (Wencker et al., 2003). Oxidative stress is enhanced in patients with heart failure (Landmesser et al., 2002). Increased ROS production leads to an imbalance between it and antioxidant defense such as vitamin E, glutathione, and antioxidant enzymes such as superoxide dismutase (SOD), catalase, and glutathione peroxidation, etc., resulting in oxidative stress (Kittleson and Hare, 2005). Mechanisms of ROS production include mitochondrial electron transport and many enzymes such as NADPH oxidase (Nox), xanthine oxidase (XO), lipoxygenase, cyclooxygenase, myeloperoxidase, etc (Zhang et al., 2012). In patients with heart failure, the expression of Nox and XO is upregulated and their activities are increased in myocardial tissues (Cappola et al., 2001; Heymes et al., 2003). There are seven members of the Nox family, including Nox1-5 and Duox1-2 (Ago et al., 2010). In heart failure, it is predominantly Nox4 that mediates ROS production, and Nox4 is located in cardiomyocytes (Kuroda et al., 2010). In heart failure mouse heart tissue, ROS production is increased (Ide et al., 1999). Increased ROS production inactivates SERCA2a leading to dysregulation of intracellular calcium homeostasis causing endoplasmic reticulum stress (Viner et al., 1996; Tagawa et al., 2008). 14,15-EET inhibits ROS production and promotes heme oxygenase-1 (HO-1) expression (Yu et al., 2015). HO-1 is an antioxidant stress enzyme that also can inhibit endoplasmic reticulum stress (Kim et al., 2011). 11,12-EET reduces ROS levels by promoting the expression of the antioxidant enzymes SOD and catalase (Liu et al., 2011).

Inflammation promotes the worsening of heart failure, and the levels of inflammatory factors are positively correlated with the severity of heart failure (Tsutsui et al., 2011). Nox4 can regulate the expression of sEH. In endothelial cells, elevated Nox inhibits sEH expression (Hu et al., 2017). However, in vascular smooth muscle cells, elevated Nox4 promotes sEH expression (Tong et al., 2016). In H9c2 cells, elevated Nox4 promoted sEH expression, and elevated sEH promoted the production of inflammatory factors CCL2 and CCL5 indirectly exerting cardiac injury (Stevenson et al., 2019). Previous studies have shown that macrophage metabolic pathways can influence macrophage function: proinflammatory macrophage (M1) production of ATP is mainly dependent on glycolysis, and anti-inflammatory macrophage (M2) production of ATP is mainly dependent on oxidative phosphorylation (Diskin and Palsson-McDermott, 2018). 14,15-EET promotes oxidative phosphorylation of macrophages, inhibits macrophage polarization towards pro-inflammatory type, and attenuates cardiac impairment due to inflammation (Ma et al., 2022).

### 1.5 Roles of other EpFAs

The previous section only discussed the role of EpFAs of AA origin, i.e., EETs, in cardiovascular system diseases. But there are other fatty acid derived EpFAs that also play important roles in cardiovascular system diseases (Kris-Etherton et al., 2002).

EPA and DHA belong to the omega-3 PUFAs (Warner et al., 2020). Eicosapentaenoic acid (EPA) and docosapentaenoic acid (DHA) are catalyzed by CYP2C and 2 J to produce epoxy-fatty acids, i.e., epoxyeicosatrienoic acids (EEQs) and epoxydocosapentaenoic acids (EDPs), and catalyzed by CYP4A and 4F to produce 20-hydroxyeicosapentaenoic acid (20-HEPE) and 22-hydroxydocosahexaenoic acid (22-HDHA), respectively (Hu et al., 2017). The half-lives of the EEQs and EDPs are very short, and they are hydrolyzed to inactive or low-activity dihydroxyeicosatetraenoic acids (DHEQs) and dihydroxyeicosatetraenoic acids (EHDPs), respectively, mainly under the sEH catalyzed hydrolysis (Figure 1) (Morisseau et al., 2010).

EEQs and EDPs function much like EETs.

First, they both lower blood pressure. Previous literature has reported that EPA and DHA have blood pressure lowering effects (Mori et al., 2000). In the angiotensin II-hypertensive mice model, EPA and DHA exerted their blood pressure-lowering effects mainly through the metabolites EEQs and EDPs (Ulu et al., 2013). The mechanism of blood pressure lowering by EEQs and EDPs is the same as that of EETs, which is mainly through the activation of KCa in vascular smooth muscle cells leading to hyperpolarization of the cell membrane, closure of the voltage-dependent calcium channel, resulting in vasodilation (Lauterbach et al., 2002; Wang R.x. et al., 2010).

Second, they both inhibit atherosclerotic plaque formation. It has been previously elaborated that endothelial cells express adhesion molecules such as P-selectin, E-selectin, VCAM-1, ICAM-1, etc., which can promote the adhesion of circulating monocytes to the surface of endothelial cells and participate in the formation of atherosclerotic plaques (Zhang et al., 2012). EETs can exert anti-atherosclerotic effects by inhibiting the activation of NF-κB and down-regulating the expression of adhesion molecules of intracellular endothelial cells (Jiang et al., 2015). 17,18-EEQ exerts anti-atherosclerotic effects by inhibiting the expression of endothelial cell ICAM-1 and E-selectin that exert anti-atherosclerotic effects (Hasegawa et al., 2017).

Third, they can both play an anti-inflammatory role. It has been shown that EETs can exert anti-inflammatory effects by inhibiting NF-κB activation and reducing the expression and release of pro-inflammatory inflammatory factors, such as TNF-a (Node et al., 1999). 19,20-EDP also exerts anti-inflammatory effects through this mechanism (Samokhvalov et al., 2015). Previous literature has reported that PPARγ activation downregulates the expression of many proinflammatory inflammatory factors (Becker et al., 2006). 17,18-EEQ can exert anti-inflammatory effects through PPARγ activation (Morin et al., 2010).

Fourth, they all have antiarrhythmic effects. It has been shown previously that EETs exert antiarrhythmic effects by modulating the activity of cardiac ion channels such as KATP, L-type Ca2+ channels, and Na + -channel (Seubert et al., 2004; Xiao, 2007). In the neonatal rat cardiomyocytes, 17,18-EEQ and 19,20-EDP inhibited the elevation of cardiomyocyte beating rates caused by elevated calcium ion concentrations, suggesting that they may have antiarrhythmic effects (Arnold et al., 2010).

## 2 Conclusion

Many scientific studies have shown that PUFAs are beneficial to human health.

AA belongs to omega-6 PUFAs (Pallas et al., 2020). AA has several metabolic pathways, such as COXs, LOXs, and CYPs pathways (Imig, 2019; Ren, 2019; Shan and Hashimoto, 2022; Zhang et al., 2022). AA generates EETs catalyzed mainly by CYP2C and 2J and 20-HETE catalyzed by CYP4A and 4F (Hoxha et al., 2020). EPA and DHA belong to omega-3 PUFAs (Ma et al., 2022). EPA and DHA are mainly catalyzed by CYP2C and 2 J to generate EEQs and EDPs, and by CYP4A and 4F to generate 20-HEPE and 22-HDHA (Fischer et al., 2014). EETs, EEQs, and EDPs belong to the EpFAs, which can be hydrolyzed to inactive or low-activity DHETs, DHEQs, and DHDPs by sEH-H catalysis (Figure 1) (Kris-Etherton et al., 2002).

**FIGURE 2:**
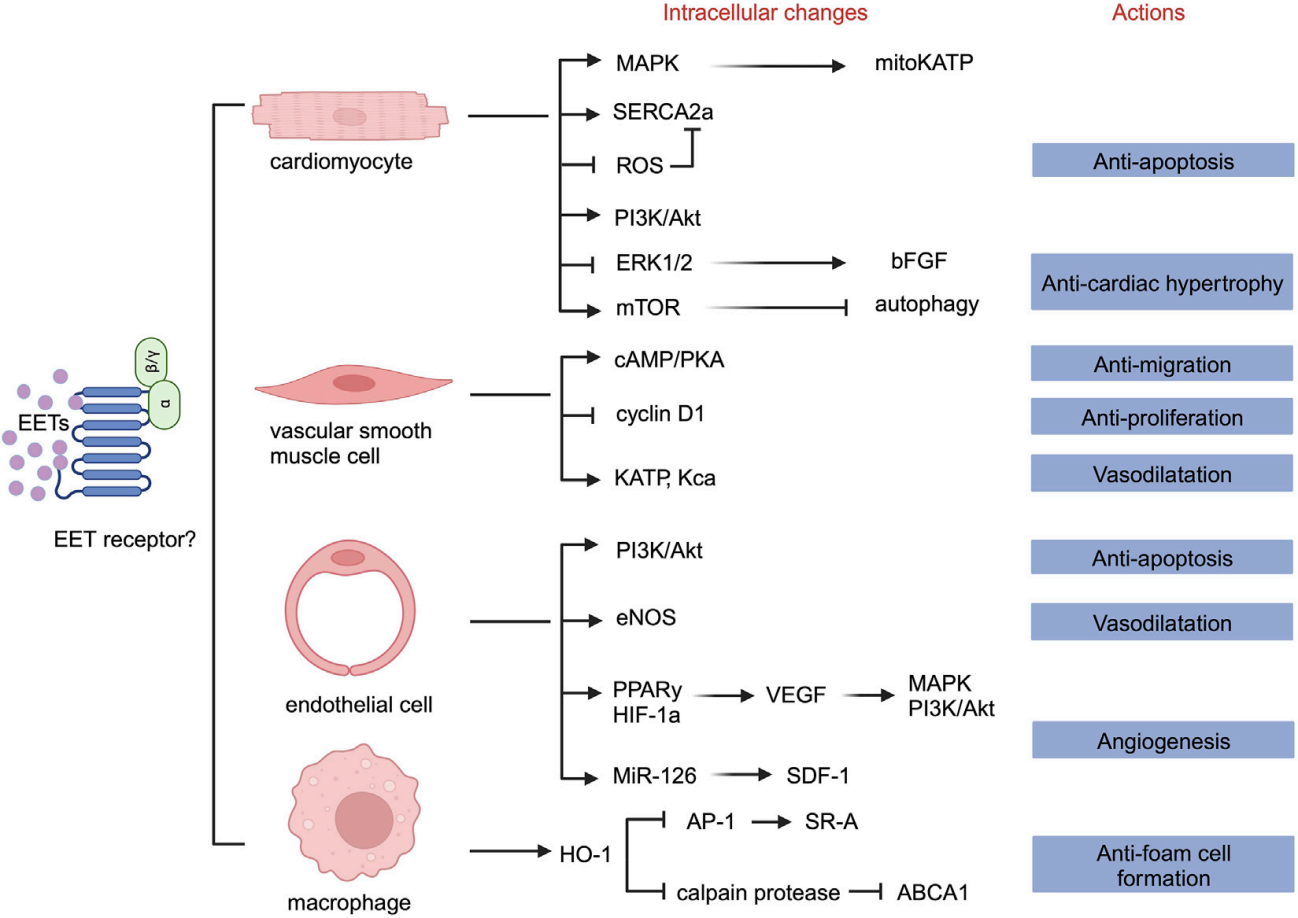
Overview of the roles of EETs in different cells.

The activity of sEH-H contributes to the onset and progression of cardiovascular diseases by promoting the hydrolysis of EETs, reducing their levels, and inhibiting their effects. We summarizes that EETs mainly play the following roles in cells constituting the cardiovascular system. First, EETs can inhibit cardiomyocyte apoptosis and hypertrophy. Second, EETs can inhibit endothelial cell apoptosis. Third, EETs can inhibit endothelial cell inflammation. Fourth, EETs can promote endothelial cell proliferation and migration, leading to angiogenesis. Fifth, EETs can promote hyperpolarization of vascular smooth muscle cells, leading to vasodilation and blood pressure reduction. Sixth, EETs can inhibit the proliferation and migration of vascular smooth muscle cells and reduce the formation of foam cells. Seventh, EETs can participate in macrophage cholesterol metabolism and reduce foam cell formation (Figure 2).

It has been reported in the literature that EEQs and EDPs also play important roles in the cardiovascular system. We conclude that EEQs and EDPs mainly play the following roles. First, EEQs and EDPs can promote the hyperpolarization of vascular smooth muscle cells, leading to vasodilation and blood pressure reduction; Second, EEQs and EDPs can inhibit monocyte adhesion to endothelial cells, and anti-atherosclerotic plaque formation; Third, EEQs and EDPs can regulate cardiac ion channels and play an anti-arrhythmic role. Fourth, EEQs and EDPs can play an anti-inflammatory role.

## 3 Discussion

sEH is a bifunctional enzyme consisting of an N-terminal sEH-P and a C-terminal sEH-H.

The function of sEH-H and sEH-P is closely related to the cell type.

In the same type of cell, sEH-H and sEH-P may have the same role. In endothelial cells, sEH-H and sEH-P act together to inhibit the expression and activity of eNOS (endothelial NO synthase) and reduce endothelial NO production (Hercule et al., 2009; Hou et al., 2012; Hou et al., 2015). The combined action of sEH-H and sEH-P inhibits the growth of human hepatoma cells (Oguro et al., 2009).

The effects of sEH-H and sEH-P are not exactly the same in different cells. In the human liver cell line (HepG2), the sEH-H and sEH-P have opposite effects, that is, sEH-P increases intracellular cholesterol levels while sEH-H decreases intracellular cholesterol levels (EnayetAllah et al., 2008). In adipocyte cell line 3T3-L1, oral administration of t-AUCB, an inhibitor of sEH-H activity, induces the upregulation of ABCA1, thereby increasing cholesterol efflux and decreasing intracellular cholesterol levels in the adipocytes, indicating that the sEH-H elevated intracellular cholesterol levels (Shen et al., 2015). In bone marrow-derived macrophages (BMDM), application of AUDA, an inhibitor of sEH-H activity, did not affect macrophage intracellular cholesterol accumulation, and application of ebselen, an inhibitor of sEH-P activity, exacerbated macrophage intracellular cholesterol accumulation, suggesting that sEH-H is not involved in BMDM cholesterol metabolism, whereas sEH-P decreased intracellular cholesterol levels (Lien et al., 2019). In THP-1, sEH-H and sEH-P act together to attenuate intracellular cholesterol accumulation in macrophages (Lien et al., 2019; Peng et al., 2019).

In different cells, sEH-H can have completely opposite effects. The sEH-H promotes the proliferation and migration of vascular smooth muscle cells but suppresses the proliferation and migration of endothelial cells (Sun et al., 2002; Davis et al., 2002; Wang et al., 2005).

So we paid close attention to the role of EETs in different cells. This is also one of the highlights of this review.

As discussed previously, in systemic circulation vessels, EETs can lower blood pressure by relaxing vascular smooth muscle cells through a variety of mechanisms (Campbell et al., 1996; Fang et al., 1999; Ye et al., 2005; Carroll et al., 2006; Kopf et al., 2011; Alkayed et al., 2022; Zhu et al., 2022). In the pulmonary circulatory vasculature, EETs exert exactly the opposite effect. *In vitro* experiments in animals have shown that EETs can cause constriction of rabbit pulmonary arteries (Zhu et al., 2000). The translocation of transient receptor potential C6 (TRPC6) from the perinuclear Golgi apparatus to the cell membrane increases the amount of TRPC6 in the cell membrane, thus exerting its function (Bezzerides et al., 2004). TRPC6 is the cellular entry channel for calcium ions that elevates intracellular calcium ion concentrations (Fleming et al., 2007). sEH inhibitors such as ACU and AEPU as well as 11,12 -EET promote the translation of TRPC6 in pulmonary artery smooth muscle cells, increasing calcium ions into the cells, leading to smooth muscle contraction and pulmonary artery pressure increase, which is detrimental to the recovery of pulmonary hypertension (Keserü et al., 2008). Thus, we conclude that both sEH and EETs are involved in the development of pulmonary hypertension, consistent with previous findings (Keserü et al., 2010).

EEQs and EDPs do not function in the same way as EETs. It has been previously discussed that EETs promote endothelial cell proliferation (Wang et al., 2005). Cyclin D1 has been shown previously to regulate the cell cycle and is negatively regulated by MAPK (Lavoie et al., 1996). 17,18-EEQ inhibits endothelial cell proliferation by downregulating cyclin D1 expression through activation of the MAPK signaling pathway (Cui et al., 2011).
